# Testing home-based exercise strategies in underserved minority cancer patients undergoing chemotherapy (THRIVE) trial: a study protocol

**DOI:** 10.3389/fonc.2024.1427046

**Published:** 2024-09-16

**Authors:** Huimin Yan, Paola Gonzalo-Encabo, Rebekah L. Wilson, Cami N. Christopher, James D. Cannon, Dong-Woo Kang, John Gardiner, Michelle Perez, Mary K. Norris, Daniel Gundersen, Laura L. Hayman, Rachel A. Freedman, Timothy R. Rebbeck, Ling Shi, Christina M. Dieli-Conwright

**Affiliations:** ^1^ Department of Exercise and Health Sciences, Manning College of Nursing and Health Sciences, University of Massachusetts Boston, Boston, MA, United States; ^2^ Division of Population Sciences, Department of Medical Oncology, Dana-Farber Cancer Institute, Boston, MA, United States; ^3^ Universidad de Alcalá, Facultad de Medicina y Ciencias de la Salud, Departamento de Ciencias Biomédicas, Área de Educación Física y Deportiva, Madrid, Spain; ^4^ Department of Medicine, Harvard Medical School, Boston, MA, United States; ^5^ Department of Nutrition, Harvard T.H. Chan School of Public Health, Boston, MA, United States; ^6^ Department of Nursing, Manning College of Nursing and Health Sciences, University of Massachusetts Boston, Boston, MA, United States; ^7^ Department of Medical Oncology, Dana-Farber Cancer Institute, Boston, MA, United States; ^8^ Department of Epidemiology, Harvard T.H. Chan School of Public Health, Boston, MA, United States

**Keywords:** cancer, racial and ethnic minorities, home-based exercise, exercise participation, cardiovascular risk factors

## Abstract

**Background:**

Higher rates of physical inactivity and comorbid conditions are reported in Hispanic/Latinx and Black cancer patients receiving chemotherapy compared to their White counterparts. Despite the beneficial effect of exercise training for cancer patients, rates of participation in exercise oncology clinical trials are low among disadvantaged and racial and ethnic minority groups. Here, we will examine the effect of an exercise intervention using a novel, accessible, and cost-effective home-based exercise approach among Hispanic/Latinx and Black cancer patients receiving chemotherapy on exercise participation and cardiovascular disease risk.

**Methods:**

The THRIVE trial is an 8-month prospective, three-arm study of 45 patients who are randomized in a 1:1:1 fashion to a supervised exercise intervention (SUP), unsupervised exercise (UNSUP), or an attention control (AC) group. Eligible patients include those with breast, colorectal, or prostate cancer, who are sedentary, overweight or obese, self-identify as Hispanic/Latinx or Black, and plan to receive chemotherapy. Patients randomized to the SUP group participate in a home-based 16-week periodized aerobic and resistance exercise program performed three days per week, supervised through video conference technology. Patients randomized to the UNSUP group participate in an unsupervised 16-week, telehealth-based, periodized aerobic and resistance exercise program performed three days per week using the same exercise prescription parameters as the SUP group. Patients randomized to the AC group receive a 16-week home-based stretching program. The primary outcome is changes in minutes of physical activity assessed by 7-day accelerometry at post-intervention. Secondary outcomes include cardiovascular risk factors, patient-reported outcomes, and physical function. Outcome measures are tested at baseline, post-intervention at month 4, and after a non-intervention follow-up period at month 8.

**Discussion:**

The THRIVE trial is the first study to employ a novel and potentially achievable exercise intervention for a minority population receiving chemotherapy. In addition, this study utilizes an intervention approach to investigate the biological and behavioral mechanisms underlying exercise participation in these cancer patients. Results will guide and inform large randomized controlled trials to test the effect of home-based exercise on treatment outcomes and comorbid disease risk in minority patients with cancer undergoing chemotherapy.

**Clinical trial registration:**

https://classic.clinicaltrials.gov/ct2/show/NCT05327452, identifier (NCT#05327452).

## 1 Introduction

Cancer is the leading cause of mortality in Hispanic/Latinx adults, and the second leading cause of death among Black adults (1–3). Particularly, obesity-related cancers, including breast, prostate, and colorectal cancer, are more prevalent among minority populations (3–5). In the U.S., the largest minority group is the Hispanic/Latinx population, comprising 60.6 million people and representing 18% of the nation’s total population (3, 6). The non-Hispanic Black population is the second largest minority population in the U.S. with 40.1 million people, accounting for approximately 12.1% of the nation’s total population (7). Chemotherapy is an essential component of treatment for many patients, administered to prevent the growth and survival of cancer cells. However, the decline in cardiorespiratory fitness paired with cardiotoxic effects associated with treatments, puts cancer patients at a higher risk for cardiovascular disease (CVD) incidence and mortality (8–10). The potential negative effects of chemotherapy may further exacerbate cardiovascular burden in Hispanic/Latinx and Black patients who are disproportionately affected by CVD. However, data on racial differences specifically in treatment-related toxicities remains scarce (11–13).

Exercise is known as a non-pharmacological tool to reduce CVD risk factors in the general population (14). Among ethnically diverse cancer patients and survivors, exercise has shown to decrease low-density lipoprotein cholesterol (15), systolic blood pressure (15, 16), the presence of diabetes (15) and body weight (16), while increasing cardioprotective high-density lipoprotein cholesterol (15, 16) and improving components of metabolic syndrome (16, 17). Further, an increasing body of literature has shown that exercise during chemotherapy is safe and feasible (18, 19), reduces cardiometabolic risk factors, and maintains or improves different health outcomes, including physical fitness, physical function, and fatigue (20–23).

**Figure 1:**
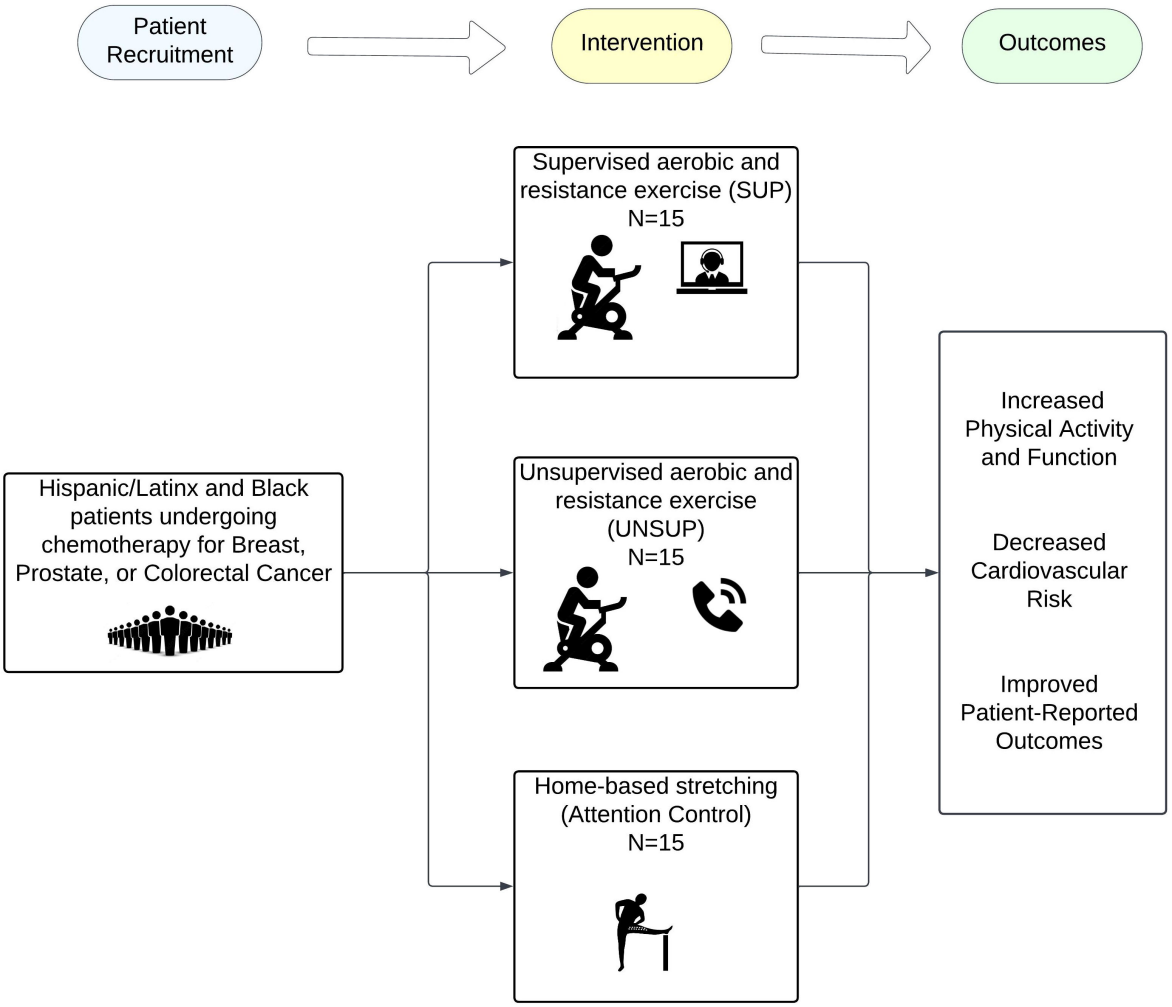
Conceptual framework of the THRIVE trial.

Despite the beneficial effects of exercise for cancer patients, racial and ethnic minority groups have been largely underrepresented in exercise clinical trials (24–26). Furthermore, minority cancer patients may face unique barriers to exercise participation and research participation, such as lack of transportation, interference with work/family responsibilities, language barriers, lack of enjoyment, etc. (27, 28) Home-based exercise interventions are more cost-effective and desired for minority cancer patients and could potentially help to overcome barriers to exercise in this population (29–32). The feasibility of educational self-directed home-based physical activity intervention (i.e., booklet with exercise recommendations) has been demonstrated in obese/overweight Hispanic/Latinx and Black adults and cancer survivors (31, 32). However, to our knowledge no study has examined the effects of home-based exercise program in minority cancer patients undergoing chemotherapy nor using a virtually supervised approach. Thus, we are currently conducting the THRIVE trial, a randomized controlled trial to determine whether virtually supervised and unsupervised home-based exercise could increase exercise participation and improve CVD risk factors among Hispanic/Latinx and Black cancer patients undergoing chemotherapy for the treatment of breast, colorectal, and prostate cancer (**Figure 1**).

## 2 Methods and analysis

### 2.1 Study design

**Figure 2:**
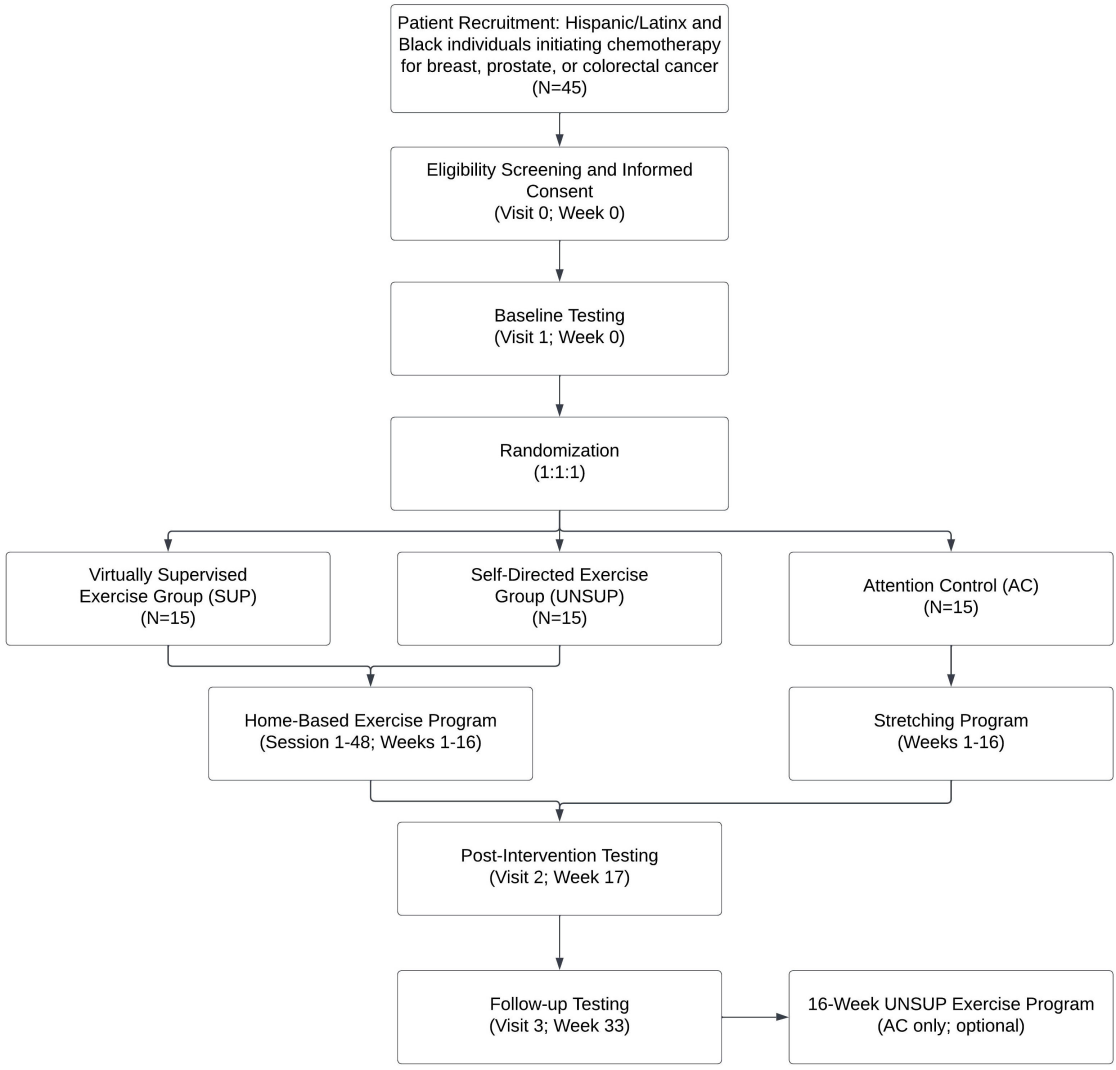
Study schema of the THRIVE Trial.

The THRIVE study is a prospective, single-center, three-arm randomized controlled trial conducted through Dana-Farber Cancer Institute in Boston, MA. The study aims to enroll 45 Hispanic/Latinx and Black patients with newly diagnosed breast, colorectal, or prostate cancer, who are receiving chemotherapy. Patients are randomized in a 1:1:1 allocation to one of three groups: (1) virtually supervised exercise intervention (SUP), (2) self-directed unsupervised exercise (UNSUP), and (3) attention control (AC). The study intervention period is 16 weeks with an additional follow-up period at 4 months post-intervention. The study protocol follows the Consolidated Standards of Reporting Trials guidelines, and the study patient flow diagram is shown in **Figure 2**. The study has been approved by the Institutional Review Board at the Dana-Farber Cancer Institute (IRB #21-559) and the University of Massachusetts Boston Institutional Review Board approved this study under a reliance agreement. The study is registered at ClinicalTrials.gov (NCT05327452).

### 2.2 Participant eligibility

This study involves Hispanic/Latinx and Black patients who have been diagnosed with breast, colorectal, or prostate cancer. Specifically, patients are eligible if they are: (1) over 18 years old; (2) diagnosed with stage I-III breast, colorectal, or prostate cancer, or metastatic prostate or colorectal cancer; (3) self-identified as Hispanic/Latinx, or Black; (4) within 4 weeks of initiating chemotherapy; (5) overweight or obese (BMI ≥25kg/m^2^ or body fat percent >30%); (6) not pregnant or planning to become pregnant during study participation; (7) cleared by their physician to participate in moderate-vigorous intensity exercise; (8) English- or Spanish-speaking; (9) currently participating in less than or equal to 60 minutes of structured moderate to vigorous exercise/week; (10) willing to travel to Dana-Farber Cancer Institute for necessary data collection; and (11) able to understand and the willingness to sign a written informed consent document. Patients are excluded if they have: (1) another active malignancy, (2) pre-existing musculoskeletal and/or cardiorespiratory conditions; (3) and/or any uncontrolled illness, including ongoing or active infection, diabetes, hypertension, or thyroid disease, or other condition that may exacerbate or be deemed unsafe with exercise.

### 2.3 Recruitment and informed consent

All patient recruitment is conducted by Dana-Farber Cancer Institute clinical research staff. To find potential participants, the study uses various recruitment strategies such as screening patient clinic lists, displaying advertisements in patient waiting areas, and promoting the study through online platforms and newsletters. Once potential participants are identified, a study coordinator contacts their treating providers or oncologists to request permission to invite them to participate in the study. Study staff then screen these participants by phone to confirm their eligibility, including their current exercise levels using the Godin Leisure Time Questionnaire (33) and their past and current medical conditions using the Physical Activity Readiness Questionnaire (PAR-Q) (34). Interested participants are scheduled for a follow-up appointment with a member of the study staff to review the protocol and sign informed consent. Electronic consent (e-consent) or wet signature is used to obtain consent.

### 2.4 Randomization and blinding

After baseline assessments are completed, patients are randomly assigned to the SUP, UNSUP, or AC group with an equal allocation ratio (1:1:1) using block randomization with a block size of 6. The randomization is stratified by cancer stage (i.e., I, II, III for breast cancer, and I, II, III, or metastatic for prostate and colorectal cancer) which is assessed at time of diagnosis. A study biostatistician (H.U.) creates the randomization schema before the trial begins, and the randomization allocation is given to staff through a web-based application (REDCap) (35). The study investigators are blind to the randomization process. The intervention allocation is not hidden from the study participants, interventionists, and outcome assessors due to the nature of exercise intervention.

### 2.5 Exercise interventions

#### 2.5.1 Virtually supervised exercise intervention (SUP group)

Patients randomized to the SUP intervention participate in a home-based 16-week periodized aerobic and resistance exercise program performed three days per week, with each session lasting approximately 45-90 minutes based on the week in the training period. SUP exercise sessions are supervised by an exercise trainer through video conference technology (i.e., Zoom). The home-based intervention consists of a systematically progressed exercise program that starts at a low intensity and gradually increases to high intensity over the 16-week duration. Each session begins with a 5 min warm-up of low-intensity aerobic cycling on a stationary bike and concludes with 5 min of cooldown. Total-body resistance training is comprised of 6 resistance exercises performed using dumbbells and resistance bands, followed by the cycling aerobic exercise bout. Heart rate (HR) is monitored throughout aerobic exercise using a Fitbit heart rate monitor. One-repetition maximum (1RM) and maximal power output (wattage) obtained during baseline testing are used to determine resistance load for each resistance exercise and aerobic intensity, respectively. The details of a single exercise session of the SUP and UNSUP programs are further depicted in **Table 1**.

**Table 1:** The THRIVE trial by intervention arm.

Intervention Arm	Supervised (SUP)	Unsupervised (UNSUP)	Attention Control (AC)
Oversight	Exercise trainer during each session via Zoom	Exercise trainer via weekly calls; sessions performed independently	Participants complete weekly records; stretching performed independently
Exercise frequency	3 sessions per week	3 sessions per week	3 sessions per week
Exercise intensity	Moderate-vigorous; monitored by maximal power output (wattage) and %RM	Moderate-vigorous; monitored by maximal power output (wattage) and %RM	Low intensity; low impact
Exercise time	45-90 min/session	45-90 min/session	10-15 min/session
Exercise type	Cycling & resistance exercise	Cycling & resistance exercise	Stretching

**Figure 3:**
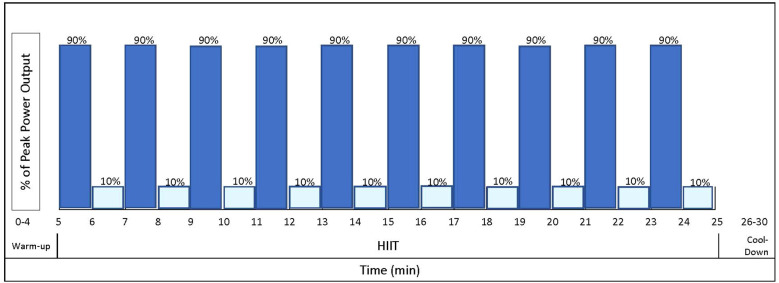
Sample high intensity interval training (HIIT) workout. Dark blue bars indicate high intensity intervals at 90% maximal power output. Light blue bars indicate one-minute rest intervals at 10% maximal power output.

The resistance exercise component utilizes a linear periodization to progress load and volume across four, one month-long mesocycles (**Table 2**), to optimally elicit improvements in body composition and physical function (36, 37). Briefly, in the first mesocycle, participants exercise at low intensity (40-45% 1RM) in an effort to increase tolerance of higher training intensities that occur later in the program. In the subsequent mesocycles, the program increases in intensity to be performed at moderate-to-vigorous intensity (70-75% 1RM) to emphasize increases in body composition and glucose metabolism (38). The volume (e.g., repetition and sets) of resistance exercises also increases throughout the intervention period (**Table 2**). The aerobic exercise component incorporates a high-intensity interval training (HIIT) program, consisting of a 20-minute stimulus period with high-intensity interval bouts at 90% maximal power output and a recovery bout at 10% at a 1-minute:1-minute (work: recovery) ratio (**Figure 3**). The ‘high’ interval is increased by 10% each week, starting from 40%, until it reaches 90%. Once 90% is reached, the intensity remains there for the remainder of the study. In the event that a participant is unable to achieve compliance with a HIIT program, a moderate-intensity interval training program will be substituted as an alternative exercise prescription to continue to provide exercise programming for participants who may be physically unable or unwilling to participate or comply fully with a HIIT program. The intensity of the moderate interval is calculated as a percentage of the individual’s estimated VO_2max_ and selected according to the highest intensity that the participant is able to tolerate/complete.

**Table 2:** Periodization model for the resistance exercise component of the exercise intervention.

Training Period	Resistance Exercise	
Mesocycle Microcycle	Intensity (%1RM)	Volume (Repetitions x Sets)
**Mesocycle 1** (Preparatory)		
Weeks 1-2	40%	10 x 3
Weeks 3-4	45%	10 x 3
**Mesocycle 2** (Moderate Intensity)		
Weeks 5-6	50%	10 – 12 x 3
Weeks 7-8	55%	10 – 12 x 3
**Mesocycle 3** (Moderate – Vigorous Intensity)		
Weeks 9-10	60%	12 x 3
Weeks 11-12	65%	12 x 3
**Mesocycle 2** (Vigorous Intensity)		
Weeks 13-14	70%	15 x 3
Weeks 15-16	75%	15 x 3

To increase compliance and aid in the standardization of the exercises, one-on-one supervision utilizing video chat (i.e., Zoom) with an exercise trainer occurs for each SUP exercise session. In addition, participants are asked to complete weekly records of physical activity performed outside the study. During each Zoom session, exercise trainers provide verbal encouragement, corrections to exercise technique, and modifications based on physical capabilities to mimic direct supervision in a clinic-based intervention. Wattage is monitored during each exercise session to ensure exercise intensity is achieved and maintained throughout each bout. Exercise equipment, including a stationary bike, resistance equipment, a Fitbit heart rate monitor, and a Wi-Fi enabled tablet, is delivered directly to each participant’s home in order to perform the intervention at home. Detailed written instructions on exercise equipment, connecting to video conferencing are provided and internet access is also provided for those who do not have access.

#### 2.5.2 Self-directed exercise intervention with regular guidance (UNSUP group)

Patients randomized to the UNSUP intervention participate in an unsupervised 16-week, telehealth-based, periodized aerobic and resistance exercise program performed three days per week using the same exercise prescription parameters as the SUP intervention (**Table 2**, **Figure 3**). Participants are provided with a stationary bike, resistance equipment, and a Fitbit heart rate monitor. HR is monitored during each exercise session using a Fitbit heart rate monitor to ensure exercise intensity is achieved and maintained throughout each bout. The exercise trainer calls each participant in the UNSUP intervention on a weekly basis to monitor progress and patients additionally complete physical activity logs. This training strategy will help to ensure that the intervention is implemented in the same way across all patients randomized to the UNSUP group. The phone calls are individualized as necessary to address individual problems to overcome barriers to success. Data on compliance and changes in physical activity reported by participants are captured and recorded electronically during the calls.

#### 2.5.3 Attention control group

The AC intervention receives a 16-week home-based stretching program, consisting of the same stretches utilized in the SUP and UNSUP interventions. The stretching protocol consists of one set of 4 static stretching exercises held for 30 seconds and performed 3 days per week. Because flexibility exercises are low-intensity, low-impact, and low-volume, minimal caloric expenditure is expected to be incurred. To increase compliance and aid in standardization, participants receive an instructional manual describing the stretches, and are instructed on the stretching exercises by an exercise trainer at baseline. Participants complete weekly records of stretching compliance and stretching adherence is captured as the percent and number of prescribed stretching sessions performed. Participants in the AC group will be asked not to change their exercise habits during the 8-month study period. Patients in the AC group are permitted to participate in a modified UNSUP exercise condition after the 8-month study period.

### 2.6 Measurements

Testing occurs at baseline (Week 0), post-intervention (Week 17), and follow-up (Week 33). All measures are completed at baseline, post-intervention, and follow-up unless otherwise specified below. A complete data collection schedule is provided in **Table 3**.

**Table 3:** Timeline of the study visits of the THRIVE Trial.

Timepoints	Eligibility Screening	Baseline Testing	Intervention	Post-Intervention Testing	Follow-up Testing
	t_0_	t_1_ Week 0	Weeks 1-16	t_2_ Week 17	t_3_ Week 33
Assessments					
Medical History	✓				
Pregnancy Test (if applicable)	✓				
Physical Activity Level		✓		✓	✓
Blood Biomarkers		✓		✓	✓
Brachial and Non-invasive Aortic Blood Pressure		✓		✓	✓
Anthropometrics		✓		✓	✓
Muscular Strength		✓		✓	✓
Cardiopulmonary Fitness		✓		✓	✓
Physical Function		✓		✓	✓
Patient-Reported Outcomes		✓		✓	✓
Dietary Recall		✓		✓	✓
Exercise or Stretching Intervention			✓		
Assessment of Adverse Events, Toxicities, or Exercise Complications			✓		
Exit Survey					✓

Arrow denotes study visits.

#### 2.6.1 Primary outcomes

Minutes of moderate-to-vigorous physical activity are utilized as the primary outcome variable and assessed via the ActiGraph wGT3X-BT (*ActiGraph, Pensacola, FL*), which is worn by patients for seven days at all times except for water activities and sleeping. Other measures of physical activity such as sedentary time, light physical activity, step count etc. are also captured using the ActiGraph. Additionally, the 7-day Physical Activity Recall interview is conducted over the phone asking participants to recall their exercise for the same 7 days that the ActiGraph was worn. Only wake wear time is utilized, and participants must have recorded a minimum of four days with at least 600 minutes of wake wear time per day for inclusion in the analysis. Non-wear time is excluded from the analysis, defined as≥90minutes of consecutive zeros with a 2-minute spike tolerance (39). Commonly used cutoff points among cancer patients will be used to classify sedentary time (<100 counts per minute), light physical activity (100–1951 counts per minute), and moderate-to-vigorous physical activity (≥1952 counts per minute) (40, 41).

#### 2.6.2 Secondary outcomes

##### 2.6.2.1 Blood biomarkers

Fasting blood is drawn by a trained phlebotomist, obtaining both serum-separating tubes and EDTA samples, which after being processed and aliquoted are stored in a -80°C freezer for later batch analysis. Blood will be analyzed through commercially available kits to assess insulin, glucose, and c-peptide, C-reactive protein, and HbA1c, where homeostasis model assessment will be used to estimate insulin resistance. These biomarkers were selected for their role as cardiovascular risk factors.

##### 2.6.2.2 Brachial and non-invasive aortic blood pressure

Brachial and aortic systolic and diastolic blood pressure are obtained non-invasively using Mobil-O-Graph (I.E.M. GmbH, Stolberg, Germany) (42). The Mobil-O-Graph is an ambulatory automated BP monitor validated for the simultaneous, non-invasive assessment of brachial and aortic blood pressure (42), and augmentation index (AIx, a marker for systemic arterial stiffness) indirectly using a mathematical transformation of the brachial pressure waveform (43). The device has been validated in healthy individuals and patients with essential hypertension (43). Measurements are taken in triplicate at rest.

##### 2.6.2.3 Anthropometrics

Height (cm), weight (kg), and waist and hip circumference (cm) are assessed according to standard procedures, with height and weight used to calculate BMI (kg/m^2^). Body composition is assessed using bioelectrical impedance (*Tanita 780, Arlington Heights, IL*), providing both appendicular and whole-body measures of fat and lean mass (kg).

##### 2.6.2.4 Cardiorespiratory fitness

To assess maximal oxygen consumption (VO_2max_), a maximal cardiopulmonary exercise test is completed on a cycle ergometer (*ErgoSelect 100, Ergoline, Germany*). After a 5-minute warm-up of no resistance, participants start at a resistance of 40 W and then proceed into an incremental ramp protocol increasing 10 W every minute until volitional fatigue (44). Cadence is kept between 60 and 70 revolutions per minute. HR (*Polar USA, Lake Success, NY*) and rate of perceived exertion (Borg scale 1-10) are recorded every minute and at the end of the test. Expired gas analysis (*TrueOne 2400, ParvoMedic, Inc., Salt Lake City, UT)* is used to measure VO_2max_. The results of this test are also used to calculate the target power output in watts for the high-intensity and recovery intervals of the HIIT program. In addition, the 6-minute Walk Test is used to estimate exercise capacity (45). Participants are instructed to walk as far as they can in 6 minutes up and down a 10-m walking course within a hallway. The distance achieved in 6 minutes will be measured in meters.

##### 2.6.2.5 Physical function

Physical function is assessed using the Short Physical Performance Battery (SPPB) (46) and sit-to-stand (47) tests. The SPPB comprises three sections: (1) balance with feet together, semi tandem, and full tandem is held for up to 10 seconds with no support (only 1 attempt is given for each position, and the time in seconds to complete it is recorded); (2) usual gait speed over 4 m is timed, where the participant has 2 attempts with the fastest time (seconds) recorded; and (3) chair stand, where the time (seconds) to complete 5 chair sit-to-stands is recorded. Each section of the SPPB is given a score dictated by performance to then provide a summary score. The sit-to-stand test involves participants completing as many sit-to-stands as possible from a seated chair position to a standing position with full hip extension in 30 seconds. The number of full movements with correct technique is recorded.

##### 2.6.2.6 Muscular strength

Muscular strength is assessed using an estimated 1-RM from 10-RM of leg press and chest press (*Matrix Fitness, Cottage Grove, WI*). Additionally, 10-RM of chair squat, chest press, glute bridge, standing row, lunges, shoulder press (i.e., the exercises completed in the exercise intervention) using resistance bands (*Bodylastics USA, Boca Raton, FL*) and dumbbells are also completed to calculate weight intensity for exercise sessions. Participants perform 1 to 2 warm-up sets of 6 to 8 repetitions. The participant proceeds to complete sets of 10 repetitions with increasing weights in each set until volitional fatigue is reached on the 10th repetition. The weight of the participant’s 10-RM is recorded in kilograms and used to estimate 1-RM using validated equations (48, 49). Isometric handgrip strength is measured in the dominant upper limb (*Camry Digital Hand Dynamometer, El Monte, CA*). Participants perform 3 trials in a standing position with their arms down by their side, with the highest result used for analysis.

##### 2.6.2.7 Psychosocial measures

Quality of life is assessed using the European Organization for Research and Treatment of Cancer questionnaires C-30 (50) for all patients, plus the cancer specific ones given to each participant based on diagnosis, prostate cancer-25 (51), colorectal cancer-29 (52), breast cancer-45 (53). Quality of life and physical function is assessed through the PROMIS-29 (54). Sleep quality is assessed via the Pittsburg Sleep Quality Index (55). Anxiety is assessed using the State Trait Anxiety Inventory (56). Common treatment-related toxicities is assessed via the patient reported outcome version of the Common Terminology Criteria for Adverse Events (57). Additionally, treatment symptoms are monitored via the therapy-related symptoms checklist completed at each supervised exercise session, and weekly phone call for the unsupervised group (58). Finally, patient views on exercise benefits, barriers, and preferences, as well as acceptability and feasibility of measures (59) are assessed in addition to their satisfaction with the intervention via an exit survey completed only at follow-up.

#### 2.6.3 Covariates

##### 2.6.3.1 Participant characteristics

Participant demographic and medical history data are collected at baseline with the administration of a study-tailored questionnaire. Changes in demographic and medical history data (e.g., medical conditions, medication, and cancer treatment) are monitored throughout the study period and confirmed at each testing time point.

##### 2.6.3.2 Dietary intake

Recent dietary patterns are assessed using the automated self-administered 24-hour dietary assessment tool (60). Participants complete 3 assessments at home on 2 weekdays and 1 weekend day, recording all food and drinks consumed during the previous 24-hour period.

##### 2.6.3.3 Physical activity logs

The AC and UNSUP groups complete a daily activity log throughout the 16-week intervention. During the supervised exercise sessions, the SUP group is asked about their physical activity completed outside the supervised sessions over the previous week. In addition, the SUP group completes daily activity logs during the follow-up period. In the activity logs, the participants are asked to record the type of exercise performed, time spent performing the exercise, intensity of the exercise bout (rate of perceived exertion [Borg scale 1-10]), and day of the week in which it was completed.

### 2.7 Intervention adherence and acceptability

Exercise adherence will be assessed according to 1) the percentage and number of prescribed sessions performed and 2) the average minutes of exercise/week, collected from the Fitbit (61). Participant adherence will be captured by 1) percentage and number of prescribed sessions attended (participants must attend≥70% of sessions to be considered compliant with exercise program e.g.,≥34 sessions), and 2) average minutes of exercise/week (participant must complete≥70% of prescribed minutes) performed at the prescribed intensity (62, 63). Intervention acceptability and feasibility will be assessed via Acceptability of Intervention and Feasibility of Intervention questionnaires at month 4 (59). Items are measured on a 5-point Likert scale (completely disagree to completely agree), and the score is calculated mean. An average rating of 4 or higher will be considered feasible/acceptable.

### 2.8 Follow-up

The planned duration of the exercise intervention is 16 weeks. However, to account for any unforeseen illness, family or medical emergency, or unplanned travel that might prevent participants from completing 48 exercise sessions within 16 weeks, all participants will have a total of 18 weeks to complete these sessions. Any sessions not completed within the 18-week period will not be made up and will be counted as a missed session. All patients will be followed for 4 months after the intervention is completed unless the patient withdraws consent. During this follow-up period, patients who are in the exercise interventions (SUP and UNSUP) will be encouraged to continue with exercise and will be provided an exercise prescription similar to the prescription provided during the exercise intervention, which will be calculated based on their post-exercise intervention assessment.

### 2.9 Adverse events

Each patient is evaluated for potential adverse events (e.g., lymphedema, pain, muscle soreness, nausea, etc.) at each supervised testing visit and each exercise session, including the supervised sessions for the SUP intervention and telehealth calls for the UNSUP intervention. Participants are provided with a phone number to contact study staff to report potential intervention-related adverse events. Participants are assessed and graded according to the National Cancer Institute Common Terminology Criteria for Adverse Events (CTCAE) V5 (64) and will be documented by the study personnel at each testing or exercise session. Staff will report on study progress and injuries during weekly meetings either in person, by phone, or through weekly email updates. All adverse events, both serious and nonserious, and deaths that are encountered from initiation of study intervention, throughout the study, and within 30 days of the last study intervention sessions, should be followed to their resolution, or until the principal investigator assesses them as stable or determines the event to be irreversible, or the participant is lost to follow-up.

### 2.10 Data monitoring and management

Data monitoring begins at the time of participant registration and will continue until protocol completion. While the study team collects, manages, and performs quality checks of the data, the principal investigator or their designated representatives may also perform quality assurance checks as needed. All data are stored on a secure network drive using REDCap (Research Electronic Data Capture; Vanderbilt University) (35, 65), a Health Insurance Portability and Accountability Act–compliant web-based application hosted by Partners HealthCare Research Computing, Enterprise Research Infrastructure & Services, on password-protected computers, with hard-copy data stored in locked filing cabinets in card-access facilities. The results of this study will be presented in publication, conference, and invited speaker formats.

### 2.11 Sample size

In order to assess change from baseline to post-intervention in daily exercise minutes for the SUP intervention versus AC; and the UNSUP intervention vs. AC, we anticipate to enroll a total of 45 patients with breast, prostate, or colorectal cancers, who will be randomized to SUP (n=15) or UNSUP (n=15) exercise groups, or AC (n=15). Based on data from a recent study led by Lee et al. (2019) examining how a HIIT intervention was feasible for breast cancer patients undergoing chemotherapy, we assume the average weekly minutes of moderate-to-vigorous physical activity to be 30 minutes for all participants at baseline, 60 minutes for the SUP and UNSUP exercise groups and 45 minutes for the AC during 4-month post-intervention, with standard deviation of 12. To detect such difference in the pre-post change in mean minutes of exercise per week (corresponding to a standardized effect size of 1.25), comparing supervised and unsupervised exercise groups to control group using t-test, with two-sided α = 0.05, a sample size of 12 subjects in each group is needed in order to achieve 80% power. Assuming 80% drop off between baseline and post-intervention (Week 17-19), a sample size of 15 patients each group is needed to be randomized.

### 2.12 Statistical analysis

Initial descriptive analyses will be used to evaluate baseline comparability among the three intervention groups, using ANOVA or Kruskal-Wallis tests for continuous variables and chi-square tests for categorical variables. We will conduct an intention-to-treat analysis for our primary and secondary endpoints. Specifically, we will conduct a t-test for the primary endpoint of difference in change in minutes of exercise for the SUP and UNSUP exercise group relative to control. The SUP and UNSUP exercise intervention will be compared to control separately. The 95% confidence interval for the mean of the difference in change in minutes of exercise will be reported. Statistical testing is for exploratory purpose, without multiple testing adjustment.

Post-intervention outcomes, including cardiovascular risk factors, patient-reported outcomes, and physical function, at 8-months will be evaluated using generalized linear mixed effects model with random intercept to adjust for clustering of repeated measurements within participants. If the primary analyses shows statistical significance, then a sensitivity analysis with multiple imputation of missing data will be conducted to test the robustness of the results. If judged to be MAR or MCAR multiple imputation will be used and between imputation variability incorporated using Rubin’s rule. We will use generalized linear mixed effects models for analyses of continuous outcomes, with appropriate distribution and link selected for any that are not normally distributed. Distribution of primary and secondary endpoints will also be examined by key socio-demographic factors (e.g. income, education level, employment status, and zip code) to explore whether these socio-demographic factors are associated with the endpoints of interest. Statistical testing will be conducted at a family-wise two-sided α = 0.05 level to determine statistical significance. All analyses will be performed using SAS (version 9.4, SAS Institute, Cary, NC).

## 3 Discussion

The THRIVE trial is the first exercise oncology trial to assess the effects of supervised and unsupervised exercise training on exercise participation in a home-based setting among physically inactive, overweight and obese Hispanic/Latinx and Black cancer patients undergoing chemotherapy. This study comprises a 16-week progressive combined resistance and HIIT intervention delivered virtually to assess exercise participation during chemotherapy, and a 16-week follow-up to evaluate the maintenance of exercise participation in the absence of exercise supervision and programming. In addition, we seek to investigate the effects of supervised and unsupervised home-based exercise training on exercise participation, cardiovascular risk factors, patient-reported outcomes, and physical function.

The THRIVE trial is the first study designed to recruit Hispanic/Latinx and Black cancer patients undergoing chemotherapy. To date, limited studies have evaluated the effect of exercise on health outcomes in Black and Hispanic/Latinx cancer survivors, with the majority of research focused on psychosocial health. Spector et al. (32) has reported improvements in weekly physical activity, cardiopulmonary fitness, muscle strength measures, functional movement, total quality of life, and fatigue in Black breast cancer survivors after completion of a home-based aerobic and resistance exercise intervention. Improvements have also been shown in quality of life, emotional well-being, social well-being, spiritual well-being, and distressed mood in both Hispanic and Black breast cancer survivors after completing a yoga intervention (66). Black men with prostate cancer undergoing androgen deprivation therapy have shown improvements in muscular strength, power, function, endurance, quality of life, and perception of fatigue following resistance only exercise (67). While no study has examined the effect of exercise training specifically in minority cancer patients undergoing chemotherapy, an increasing body of literature reveals that exercise completed during chemotherapy is not only safe, but can maintain and/or improve physical fitness, physical function, and activities of daily living. Exercise also shows potential for reducing toxicities associated with chemotherapy, including fatigue and peripheral neuropathy (20, 68). Given the demonstrated benefits and lack of clinical trials in this population, we focused on underserved minority cancer patients to bridge the gap in cancer health research in minority cancer patients undergoing chemotherapy with limited access to exercise training facilities and support.

The primary outcome of this study is physical activity levels measured by accelerometry. Physical activity participation is an important modifiable factor to improve cancer survivorship (69). Therapeutic interventions that prevent or retard the decline of physical function offer clinical benefits in cancer patients (70). Considering the reported barriers to physical activity in minority cancer survivors (71), exercise participation is a key outcome to be evaluated with exercise interventions. Increased physical activity levels have been reported in Hispanic (72, 73), and Black cancer survivors (32, 73, 74) following exercise interventions ranging between 8 to 16 weeks, suggesting the potential effectiveness of these interventions to increase exercise participation. Notably, previous studies predominantly relied on questionnaires to assess physical activity, acknowledging the higher cost and logistical complexities associated with wearable devices like accelerometers. However, self-reported physical activity data might not accurately portray participants’ activity levels, particularly before the exercise intervention, possibly due to unfamiliarity with the assessment instrument and reporting bias. Therefore, the forthcoming findings from THRIVE trial are expected to offer valuable objective measures of physical activity levels in cancer patients after an exercise intervention.

We selected cardiovascular risk factors as our secondary outcome because the potential negative effects of anticancer therapy on cardiovascular health. This may further exacerbate cardiovascular burden in Hispanic/Latinx and Black cancer patients, who are disproportionately affected by cardiometabolic diseases (10). CVD-specific mortality has been reported higher among younger Black breast cancer survivors than White breast cancer survivors (13.3% vs 8.9%, respectively) (10). Exercise is a safe, cost-effective (75), non-pharmacological approach that has been shown to reduce cardiovascular risk factors in ethnically diverse cancer patients and survivors (15–17). In addition to the traditional cardiovascular risk factors, our study will provide novel data on aortic blood pressure dynamics. Aortic blood pressure is more prognostic than conventional brachial blood pressure as aortic pressure more aptly reflects the load encountered by the heart (i.e., ventricular-vascular coupling) (76). Thus, brachial blood pressure may neglect important information on the cardiovascular burden and response to therapy in Blacks and Hispanics. The THRIVE trial will be the first to evaluate the effect of the interventions in both traditional brachial blood pressure and novel aortic blood pressure. It will also provide ancillary data on arterial stiffness to explore the potential effects on vascular function.

We have implemented novel home-based exercise training strategies to lower barriers to exercise participation in our target populations. Some reported barriers to physical activity participation include the lack of family-care support, limited access to exercise facilities, and the monetary cost of gym membership among Black and Hispanic/Latinx populations (29). Moreover, cancer patients undergoing chemotherapy experience disease- and treatment-related side effects (77), which may further lower the patients’ motivation to attend in-person exercise sessions.

Careful considerations were made in the THRIVE trial to ensure accessibility for underserved minority patients residing in the Greater Boston area. Specifically, home-based exercise is utilized in both exercise intervention groups in the THRIVE study, with the primary distinction lies in the level of supervision they receive. In addition, exercise equipment is delivered to participants’ homes and patients are provided with detailed written instructions on exercise equipment, connecting to video conferencing, and internet access (e.g., providing internet-enabled tablet for those without access).

Although home-based exercise interventions have previously been utilized in cancer survivors, the THRIVE study is the first to utilize self-directed unsupervised and virtually supervised home-based exercise interventions in minority cancer patients receiving chemotherapy. Only one study, to our knowledge, examined the feasibility and safety of home-based exercise interventions in cancer patients during chemotherapy (78). While exercise adherence of 87.6% was reported during the self-directed unsupervised exercise intervention with weekly phone calls, Sturgeon et al. (78) reported limited tolerability for the high-intensity component of their exercise dose. In a Swedish cohort of breast cancer patients undergoing chemotherapy, HIIT provided an effective and time-saving training strategy resulting in beneficial preservation of cardiorespiratory fitness (79). Similarly, Lee et al. (80) found supervised, in-clinic HIIT to be feasible and have an adherence rate of 82% in a cohort of mostly Hispanic cancer patients undergoing chemotherapy. The THRIVE trial takes the next step of exploring the feasibility of delivering a HITT exercise program remotely to cancer patients during chemotherapy. Even though the THRIVE trial is not designed to directly compare the effectiveness of self-directed unsupervised and virtually supervised exercise intervention groups, our preliminary findings will offer valuable insights for the design of future exercise trials involving cancer patients during chemotherapy.

A major strength of the THRIVE trial is the employment of tailored exercise interventions for Hispanic/Latinx and Black cancer patients during chemotherapy. Our exercise intervention is rigorously developed incorporating individualized progressive HIIT and resistance training. We also provide novel real-time supervision during home-based exercise in the virtually supervised exercise group. This model has the potential to be utilized for other cancer diagnoses among minority patients. Moreover, we are examining integration of physiological variables for the intervention to provide mechanistic information on the effectiveness of the trial. One limitation of the THRIVE trial is the lack of detailed focus on social determinants of health. However, we are addressing this to some extent by collecting participants’ income, education, employment status and zip codes at basement (T1). Performing analyses on this data will provide preliminary insights into the impact of social determinants of health and help guide the design of a larger trial in the future. Another limitation of the THRIVE trial is the inclusion of three distinct cancer types in various stages and there are gender differences in cancer occurrence. However, based on our hypothesis, we expect to see increases in physical activity levels regardless of the participants’ cancer diagnoses and staging. The preliminary data from the current trial will be used to guide and inform the design of a Phase II clinical trial with specific cancer diagnoses to assess the effects of home-based exercise interventions on cardiovascular risk in cancer patients undergoing chemotherapy and the overall efficacy of chemotherapy.

In conclusion, the THRIVE trial is the first study to employ a novel and potentially achievable exercise intervention for a minority population receiving chemotherapy. In addition, this study utilizes an intervention approach to investigate the biological and behavioral mechanisms underlying exercise participation in these cancer patients. Results from this study will guide and inform large randomized controlled trials to test the effect of home-based exercise on treatment outcomes and comorbid disease risk in minority cancer patients undergoing chemotherapy.

## References

[1] Siegel RL Miller KD Wagle NS Jemal A . Cancer statistics, 2023. CA: A Cancer J Clin. (2023) 73:17–48. doi: 10.3322/caac.21763 36633525

[2] Giaquinto AN Miller KD Tossas KY Winn RA Jemal A Siegel RL . Cancer statistics for african american/black people 2022. CA: A Cancer J Clin. (2022) 72:202–29. doi: 10.3322/caac.21718 35143040

[3] Miller KD Ortiz AP Pinheiro PS Bandi P Minihan A Fuchs HE . Cancer statistics for the US Hispanic/Latino population, 2021. CA: Cancer J Clin. (2021) 71:466–87. doi: 10.3322/caac.21695 34545941

[4] Brown JC Carson TL Thompson HJ Agurs-Collins T . The triple health threat of diabetes, obesity, and cancer—Epidemiology, disparities, mechanisms, and interventions. Obesity. (2021) 29:954–9. doi: 10.1002/oby.23161 PMC8152945 34029445

[5] DeSantis CE Miller KD Goding Sauer A Jemal A Siegel RL . Cancer statistics for african americans, 2019. CA Cancer J Clin. (2019) 69:211–33. doi: 10.3322/caac.21555 30762872

[6] Ortman JM Velkoff VA Hogan H . An aging nation: the older population in the United States. United States Census Bureau Suitland, MD (2014).

[7] U.S. Department of Health and Human Services . Black/african american (2023). Available online at: https://minorityhealth.hhs.gov/omh/browse.aspx?lvl=3&lvlid=61 (Accessed August 1, 2023).

[8] Caron J Nohria A . Cardiac toxicity from breast cancer treatment: can we avoid this? Curr Oncol Rep. (2018) 20:61. doi: 10.1007/s11912-018-0710-1 29876677

[9] Visvikis A Kyvelou SM Pietri P Georgakopoulos C Manousou K Tousoulis D . Cardiotoxic profile and arterial stiffness of adjuvant chemotherapy for colorectal cancer. Cancer Manag Res. (2020) 12:1175–85. doi: 10.2147/CMAR PMC7025666 32104097

[10] Troeschel AN Liu Y Collin LJ Bradshaw PT Ward KC Gogineni K . Race differences in cardiovascular disease and breast cancer mortality among US women diagnosed with invasive breast cancer. Int J Epidemiol. (2019) 48:1897–905. doi: 10.1093/ije/dyz108 31155644

[11] Han HS Reis IM Zhao W Kuroi K Toi M Suzuki E . Racial differences in acute toxicities of neoadjuvant or adjuvant chemotherapy in patients with early-stage breast cancer. Eur J Cancer. (2011) 47:2537–45. doi: 10.1016/j.ejca.2011.06.027 21741825

[12] Litvak A Batukbhai B Russell SD Tsai HL Rosner GL Jeter SC . Racial disparities in the rate of cardiotoxicity of HER2-targeted therapies among women with early breast cancer. Cancer. (2018) 124:1904–11. doi: 10.1002/cncr.31260 PMC5910274 29381193

[13] Patel TA Colon-Otero G Bueno Hume C Copland JA 3rd Perez EA . Breast cancer in Latinas: gene expression, differential response to treatments, and differential toxicities in Latinas compared with other population groups. Oncologist. (2010) 15:466–75. doi: 10.1634/theoncologist.2010-0004 PMC3227981 20427382

[14] Joyner MJ Green DJ . Exercise protects the cardiovascular system: effects beyond traditional risk factors. J Physiol. (2009) 587:5551–8. doi: 10.1113/jphysiol.2009.179432 PMC2805367 19736305

[15] Lee K Tripathy D Demark-Wahnefried W Courneya KS Sami N Bernstein L . Effect of aerobic and resistance exercise intervention on cardiovascular disease risk in women with early-stage breast cancer: A randomized clinical trial. JAMA Oncol. (2019) 5:710–4. doi: 10.1001/jamaoncol.2019.0038 PMC6512455 30920602

[16] Dieli-Conwright CM Courneya KS Demark-Wahnefried W Sami N Lee K Buchanan TA . Effects of aerobic and resistance exercise on metabolic syndrome, sarcopenic obesity, and circulating biomarkers in overweight or obese survivors of breast cancer: A randomized controlled trial. J Clin Oncol. (2018) 36:875–83. doi: 10.1200/JCO.2017.75.7526 PMC5858524 29356607

[17] Brown JC Rickels MR Troxel AB Zemel BS Damjanov N Ky B . Dose-response effects of exercise on insulin among colon cancer survivors. Endocr Relat Cancer. (2018) 25:11–9. doi: 10.1530/ERC-17-0377 PMC5736434 29018055

[18] Hatlevoll I Oldervoll LM Wibe A Stene GB Stafne SN Hofsli E . Physical exercise during adjuvant chemotherapy for colorectal cancer-a non-randomized feasibility study. Support Care Cancer. (2021) 29:2993–3008. doi: 10.1007/s00520-020-05789-z 33030598 PMC8062327

[19] De Lazzari N Niels T Tewes M . Götte M. A systematic review of the safety, feasibility and benefits of exercise for patients with advanced cancer. Cancers (Basel). (2021) 13:4478. doi: 10.3390/cancers13174478 34503288 PMC8430671

[20] Cave J Paschalis A Huang CY West M Copson E Jack S . A systematic review of the safety and efficacy of aerobic exercise during cytotoxic chemotherapy treatment. Support Care Cancer. (2018) 26:3337–51. doi: 10.1007/s00520-018-4295-x 29936624

[21] Schoot GGFVD Ormel HL Westerink N-DL May AM Elias SG Hummel YM . Optimal timing of a physical exercise intervention to improve cardiorespiratory fitness. JACC: CardioOncology. (2022) 4:491–503. doi: 10.1016/j.jaccao.2022.07.006 36444224 PMC9700259

[22] Van Vulpen JK Velthuis MJ Steins Bisschop CN Travier N Van Den Buijs BJ Backx FJ . Effects of an exercise program in colon cancer patients undergoing chemotherapy. Med Sci Sports Exerc. (2016) 48:767–75. doi: 10.1249/MSS.0000000000000855 26694846

[23] Gonzalo-Encabo P Christopher CN Lee K Normann AJ Yunker AG Norris MK . High-intensity interval training improves metabolic syndrome in women with breast cancer receiving Anthracyclines. Scandinavian J Med Sci Sports. (2023) 33:475–84. doi: 10.1111/sms.14280 36427275

[24] Bandera EV Alfano CM Qin B Kang D-W Friel CP Dieli-Conwright CM . Harnessing nutrition and physical activity for breast cancer prevention and control to reduce racial/ethnic cancer health disparities. Am Soc Clin Oncol Educ Book. (2021) 41):e62–78. doi: 10.1200/EDBK_321315 33989021

[25] Norris MK Fox FS Lee C Wang E Green K Yan H . Narrowing the gap for minority cancer survivors: exercise oncology in the past, present, and future. J Clin Exercise Physiol. (2021) 9:155–70. doi: 10.31189/2165-7629-9.4.155

[26] Gonzalo-Encabo P Sami N Wilson RL Kang DW Ficarra S Dieli-Conwright CM . Exercise as medicine in cardio-oncology: reducing health disparities in hispanic and latina breast cancer survivors. Curr Oncol Rep. (2023) 25:1237–45. doi: 10.1007/s11912-023-01446-w PMC10640421 37715884

[27] Brown DR Fouad MN Basen-Engquist K Tortolero-Luna G . Recruitment and retention of minority women in cancer screening, prevention, and treatment trials. Ann Epidemiol. (2000) 10:S13–21. doi: 10.1016/S1047-2797(00)00197-6 11189088

[28] Spector D Battaglini C Groff D . Perceived exercise barriers and facilitators among ethnically diverse breast cancer survivors. Oncol Nurs Forum. (2013) 40:472–80. doi: 10.1188/13.ONF.472-480 PMC10589869 23989021

[29] Richter DL Wilcox S Greaney ML Henderson KA Ainsworth BE . Environmental, policy, and cultural factors related to physical activity in African American women. Women Health. (2002) 36:91–109. doi: 10.1300/J013v36n02_07 12487143

[30] Pekmezi D Ainsworth C Joseph R Bray MS Kvale E Isaac S . Rationale, design, and baseline findings from HIPP: A randomized controlled trial testing a home-based, individually-tailored physical activity print intervention for African American women in the Deep South. Contemp Clin Trials. (2016) 47:340–8. doi: 10.1016/j.cct.2016.02.009 PMC4821007 26944022

[31] Pekmezi D Ainsworth C Joseph RP Williams V Desmond R Meneses K . Pilot trial of a home-based physical activity program for african american women. Med Sci Sports Exerc. (2017) 49:2528–36. doi: 10.1249/MSS.0000000000001370 PMC5688013 28704343

[32] Spector D Deal AM Amos KD Yang H Battaglini CL . A pilot study of a home-based motivational exercise program for African American breast cancer survivors: clinical and quality-of-life outcomes. Integr Cancer Ther. (2014) 13:121–32. doi: 10.1177/1534735413503546 PMC10568972 24105359

[33] Godin G Shephard R . Godin leisure-time exercise questionnaire. Med Sci Sports Exerc. (1997) 29:36–8. doi: 10.1097/00005768-199706001-00009

[34] Bredin SS Gledhill N Jamnik VK Warburton DE . PAR-Q+ and ePARmed-X+: new risk stratification and physical activity clearance strategy for physicians and patients alike. Can Fam Physician. (2013) 59:273–7. PMC3596208 23486800

[35] Harris PA Taylor R Minor BL Elliott V Fernandez M O'Neal L . The REDCap consortium: Building an international community of software platform partners. J BioMed Inform. (2019) 95:103208. doi: 10.1016/j.jbi.2019.103208 31078660 PMC7254481

[36] Hacker ED Collins E Park C Peters T Patel P Rondelli D . Strength training to enhance early recovery after hematopoietic stem cell transplantation. Biol Blood Marrow Transplantation. (2017) 23:659–69. doi: 10.1016/j.bbmt.2016.12.637 28042020

[37] Fiuza-Luces C Berger N Lucia A Simpson R Ramirez M Garatachea N . Understanding graft-versus-host disease. Preliminary findings regarding the effects of exercise in affected patients. Exerc Immunol Rev. (2015) 21:80–112. 25826127

[38] Kang D-W Lee J Suh S-H Ligibel J Courneya KS Jeon JY . Effects of exercise on insulin, IGF axis, adipocytokines, and inflammatory markers in breast cancer survivors: a systematic review and meta-analysis. Cancer Epidemiology Biomarkers Prev. (2017) 26:355–65. doi: 10.1158/1055-9965.EPI-16-0602 27742668

[39] Choi L Liu Z Matthews CE Buchowski MS . Validation of accelerometer wear and nonwear time classification algorithm. Med Sci Sports Exerc. (2011) 43:357–64. doi: 10.1249/MSS.0b013e3181ed61a3 PMC3184184 20581716

[40] Freedson PS Melanson E Sirard J . Calibration of the computer science and applications, inc. accelerometer. Med Sci Sports Exerc. (1998) 30:777–81. doi: 10.1097/00005768-199805000-00021 9588623

[41] Peddle-McIntyre CJ Cavalheri V Boyle T McVeigh JA Jeffery E Lynch BM . A review of accelerometer-based activity monitoring in cancer survivorship research. Med Sci Sports Exerc. (2018) 50:1790–801. doi: 10.1249/MSS.0000000000001644 29683922

[42] Franssen PM Imholz BP . Evaluation of the Mobil-O-Graph new generation ABPM device using the ESH criteria. Blood Press Monit. (2010) 15:229–31. doi: 10.1097/MBP.0b013e328339be38 20658764

[43] Weiss W Gohlisch C Harsch-Gladisch C Tolle M Zidek W van der Giet M . Oscillometric estimation of central blood pressure: validation of the Mobil-O-Graph in comparison with the SphygmoCor device. Blood Press Monit. (2012) 17:128–31. doi: 10.1097/MBP.0b013e328353ff63 22561735

[44] Jones LW Eves ND Haykowsky M Joy AA Douglas PS . Cardiorespiratory exercise testing in clinical oncology research: systematic review and practice recommendations. Lancet Oncol. (2008) 9:757–65. doi: 10.1016/S1470-2045(08)70195-5 18672211

[45] Schmidt K Vogt L Thiel C Jäger E Banzer W . Validity of the six-minute walk test in cancer patients. Int J Sports Med. (2013) 34:631–6. doi: 10.1055/s-00000028 23444095

[46] Guralnik JM Simonsick EM Ferrucci L Glynn RJ Berkman LF Blazer DG . A short physical performance battery assessing lower extremity function: association with self-reported disability and prediction of mortality and nursing home admission. J Gerontol. (1994) 49:M85–94. doi: 10.1093/geronj/49.2.M85 8126356

[47] Jones CJ Rikli RE Beam WC . A 30-s chair-stand test as a measure of lower body strength in community-residing older adults. Res Q Exerc Sport. (1999) 70:113–9. doi: 10.1080/02701367.1999.10608028 10380242

[48] Brzycki M . Strength testing—Predicting a one-rep max from reps-to-fatigue. J Phys Education Recreation Dance. (1993) 64:88–90. doi: 10.1080/07303084.1993.10606684

[49] Mayhew JL Ball TE Arnold MD Bowen JC . Relative muscular endurance performance as a predictor of bench press strength in college men and women. J Strength Conditioning Res. (1992) 6:200–6. doi: 10.1519/1533-4287(1992)006<0200:RMEPAA>2.3.CO;2

[50] Aaronson NK Ahmedzai S Bergman B Bullinger M Cull A Duez NJ . The European Organization for Research and Treatment of Cancer QLQ-C30: a quality-of-life instrument for use in international clinical trials in oncology. J Natl Cancer Inst. (1993) 85:365–76. doi: 10.1093/jnci/85.5.365 8433390

[51] van Andel G Bottomley A Fosså SD Efficace F Coens C Guerif S . An international field study of the EORTC QLQ-PR25: a questionnaire for assessing the health-related quality of life of patients with prostate cancer. Eur J Cancer. (2008) 44:2418–24. doi: 10.1016/j.ejca.2008.07.030 18774706

[52] van der Hout A Neijenhuijs KI Jansen F van Uden-Kraan CF Aaronson NK Groenvold M . Measuring health-related quality of life in colorectal cancer patients: systematic review of measurement properties of the EORTC QLQ-CR29. Support Care Cancer. (2019) 27:2395–412. doi: 10.1007/s00520-019-04764-7 PMC6541702 30982095

[53] Bjelic-Radisic V Cardoso F Cameron D Brain E Kuljanic K da Costa RA . An international update of the EORTC questionnaire for assessing quality of life in breast cancer patients: EORTC QLQ-BR45. Ann Oncol. (2020) 31:283–8. doi: 10.1016/j.annonc.2019.10.027 31959345

[54] Jensen RE Potosky AL Reeve BB Hahn E Cella D Fries J . Validation of the PROMIS physical function measures in a diverse US population-based cohort of cancer patients. Qual Life Res. (2015) 24:2333–44. doi: 10.1007/s11136-015-0992-9 PMC5079641 25935353

[55] Buysse DJ Reynolds CF 3rd Monk TH Berman SR Kupfer DJ . The Pittsburgh Sleep Quality Index: a new instrument for psychiatric practice and research. Psychiatry Res. (1989) 28:193–213. doi: 10.1016/0165-1781(89)90047-4 2748771

[56] Kayikcioglu O Bilgin S Seymenoglu G Deveci A . State and trait anxiety scores of patients receiving intravitreal injections. BioMed Hub. (2017) 2:1–5. doi: 10.1159/000478993 PMC6945947 31988910

[57] Basch E Reeve BB Mitchell SA Clauser SB Minasian LM Dueck AC . Development of the National Cancer Institute's patient-reported outcomes version of the common terminology criteria for adverse events (PRO-CTCAE). J Natl Cancer Inst. (2014) 106. doi: 10.1093/jnci/dju244 PMC4200059 25265940

[58] Williams P Ducey K Williams A Sears A Tobin-Rumelhart S Bunde P . A Therapy-Related Symptoms Checklist (TRSC) for oncology patients: a self-report instrument. Oncol Nurs Forum. (1997). doi: 10.1037/t21309-000

[59] Weiner BJ Lewis CC Stanick C Powell BJ Dorsey CN Clary AS . Psychometric assessment of three newly developed implementation outcome measures. Implement Sci. (2017) 12:108. doi: 10.1186/s13012-017-0635-3 28851459 PMC5576104

[60] Park Y Dodd KW Kipnis V Thompson FE Potischman N Schoeller DA . Comparison of self-reported dietary intakes from the Automated Self-Administered 24-h recall, 4-d food records, and food-frequency questionnaires against recovery biomarkers. Am J Clin Nutr. (2018) 107:80–93. doi: 10.1093/ajcn/nqx002 29381789 PMC5972568

[61] Kampshoff CS Jansen F Van Mechelen W May AM Brug J Chinapaw MJ . Determinants of exercise adherence and maintenance among cancer survivors: a systematic review. Int J Behav Nutr Phys Activity. (2014) 11:1–13. doi: 10.1186/1479-5868-11-80 PMC4096543 24989069

[62] Jensen W Baumann FT Stein A Bloch W Bokemeyer C de Wit M . Exercise training in patients with advanced gastrointestinal cancer undergoing palliative chemotherapy: a pilot study. Support Care Cancer. (2014) 22:1797–806. doi: 10.1007/s00520-014-2139-x 24531742

[63] Pinto BM Rabin C Dunsiger S . Home-based exercise among cancer survivors: adherence and its predictors. Psychooncology. (2009) 18:369–76. doi: 10.1002/pon.1465 PMC2958525 19242921

[64] US Department of Health and Human Services NIoH National Cancer Institute . Common terminology criteria for adverse events (CTCAE) version 5.0. US Department of Health and Human Services NIoH and National Cancer Institute. Bethesda, Maryland (2017).

[65] Harris PA Taylor R Thielke R Payne J Gonzalez N Conde JG . Research electronic data capture (REDCap)–a metadata-driven methodology and workflow process for providing translational research informatics support. J BioMed Inform. (2009) 42:377–81. doi: 10.1016/j.jbi.2008.08.010 PMC2700030 18929686

[66] Moadel AB Shah C Wylie-Rosett J Harris MS Patel SR Hall CB . Randomized controlled trial of yoga among a multiethnic sample of breast cancer patients: effects on quality of life. J Clin Oncol. (2007) 25:4387–95. doi: 10.1200/JCO.2006.06.6027 17785709

[67] Hanson ED Sheaff AK Sood S Ma L Francis JD Goldberg AP . Strength training induces muscle hypertrophy and functional gains in black prostate cancer patients despite androgen deprivation therapy. Journals Gerontology Ser A: Biomed Sci Med Sci. (2013) 68:490–8. doi: 10.1093/gerona/gls206 PMC3593619 23089339

[68] Scott JM Lee J Herndon JE Michalski MG Lee CP O'Brien KA . Timing of exercise therapy when initiating adjuvant chemotherapy for breast cancer: a randomized trial. Eur Heart J. (2023) 44:4878–89. doi: 10.1093/eurheartj/ehad085 PMC10702461 36806405

[69] Holmes MD Chen WY Feskanich D Kroenke CH Colditz GA . Physical activity and survival after breast cancer diagnosis. JAMA. (2005) 293:2479–86. doi: 10.1001/jama.293.20.2479 15914748

[70] Scott JM Nilsen TS Gupta D Jones LW . Exercise therapy and cardiovascular toxicity in cancer. Circulation. (2018) 137:1176–91. doi: 10.1161/CIRCULATIONAHA.117.024671 PMC6028047 29530893

[71] Oyekanmi G Paxton RJ . Barriers to physical activity among African American breast cancer survivors. Psychooncology. (2014) 23:1314–7. doi: 10.1002/pon.3527 PMC4167984 24644092

[72] Mama SK Song J Ortiz A Tirado-Gomez M Palacios C Hughes DC . Longitudinal social cognitive influences on physical activity and sedentary time in Hispanic breast cancer survivors. Psychooncology. (2017) 26:214–21. doi: 10.1002/pon.4026 PMC4879102 26602701

[73] Rossi A Garber CE Ortiz M Shankar V Goldberg GL Nevadunsky NS . Feasibility of a physical activity intervention for obese, socioculturally diverse endometrial cancer survivors. Gynecol Oncol. (2016) 142:304–10. doi: 10.1016/j.ygyno.2016.05.034 27246303

[74] Wilson DB Porter JS Parker G Kilpatrick J . Anthropometric changes using a walking intervention in African American breast cancer survivors: a pilot study. Prev Chronic Dis. (2005) 2(2):A16. PMC1327710 15888227

[75] Lee K Kang I Mortimer JE Sattler F Mack WJ Fitzsimons LA . Effects of high-intensity interval training on vascular function in breast cancer survivors undergoing anthracycline chemotherapy: design of a pilot study. BMJ Open. (2018) 8:e022622. doi: 10.1136/bmjopen-2018-022622 PMC6042553 29961039

[76] McEniery CM Yasmin N McDonnell B Munnery M Wallace SM Rowe CV . Central pressure: variability and impact of cardiovascular risk factors: the Anglo-Cardiff Collaborative Trial II. Hypertension. (2008) 51:1476–82. doi: 10.1161/HYPERTENSIONAHA.107.105445 18426997

[77] Gildea GC Spence RR Jones TL Turner JC Macdonald ER Hayes SC . Barriers, facilitators, perceptions and preferences influencing physical activity participation, and the similarities and differences between cancer types and treatment stages - A systematic rapid review. Prev Med Rep. (2023) 34:102255. doi: 10.1016/j.pmedr.2023.102255 37273528 PMC10236469

[78] Sturgeon KM Smith AM Federici EH Kodali N Kessler R Wyluda E . Feasibility of a tailored home-based exercise intervention during neoadjuvant chemotherapy in breast cancer patients. BMC Sports Sci Med Rehabil. (2022) 14:31. doi: 10.1186/s13102-022-00420-6 35216638 PMC8874298

[79] Mijwel S Backman M Bolam KA Olofsson E Norrbom J Bergh J . Highly favorable physiological responses to concurrent resistance and high-intensity interval training during chemotherapy: the OptiTrain breast cancer trial. Breast Cancer Res Treat. (2018) 169:93–103. doi: 10.1007/s10549-018-4663-8 29349712 PMC5882634

[80] Lee K Kang I Mack WJ Mortimer J Sattler F Salem G . Feasibility of high intensity interval training in patients with breast Cancer undergoing anthracycline chemotherapy: a randomized pilot trial. BMC Cancer. (2019) 19:653. doi: 10.1186/s12885-019-5887-7 31269914 PMC6610838

